# A Single-Center Experience: What is the Effect of Sleeve Gastrectomy in Patients With a BMI ≥ 50 kg/m²?

**DOI:** 10.7759/cureus.27992

**Published:** 2022-08-14

**Authors:** Alper Ozturk, Yusuf Celik

**Affiliations:** 1 Department of General Surgery, Biruni University, Istanbul, TUR; 2 Department of Biostatistics and Medical Informatics, Biruni University, Istanbul, TUR

**Keywords:** bariatric surgery, success rate, patients with a bmi ≥ 50, weight loss, sleeve gastrectomy

## Abstract

Background: Our research aimed to see how sleeve gastrectomy (SG) affects weight loss and comorbidities in patients with a body mass index (BMI) ≥ 50 kg/m².

Materials and methods: Prospectively kept data of patients with a BMI ≥50 kg/m² who underwent SG between February 2016 and February 2020 were evaluated.

Results: A total of 138 patients with a BMI ≥ 50 kg/m² were operated on. The average BMI was 56.36±7.661, the average age was 37.41±12.33. Forty-eight patients underwent concomitant cholecystectomy and/or hiatal hernia repair (HHR). The percentage of excess weight loss (EWL%) of patients at the 3rd, 6th, 12th, 18th, and 24th months were 36%, 54%, 67%, 72%, and 74%, respectively. Mean BMI values of the 0th, 3rd, 6th, 12th, 18th, and 24th months were 56, 45, 39, 35, 33, and 33, respectively. 0th, 3rd, 6th, 12th, 18th, and 24th months were significantly different for EWL%, total weight loss (TWL%), and BMI variables (p<0.001), but EWL% (p=0.527), TWL% (p=0.396) and BMI (p=0,657) were not found significantly different between the 18th and 24th months. When EWL% ≥ 50 was accepted, the success rate was 93% (n=93) and 92% (n=50) at the 18th and 24th months, respectively. While there was 82% remission in type 2 diabetes mellitus (DM) and 90% in hypertension (HT), the remission rate in patients with obstructive sleep apnea syndrome (OSAS) and gastroesophageal reflux disease (GERD) undergoing HHR was 100%.

Conclusions: In patients with a BMI ≥ 50 kg/m², SG seems to be an effective and safe therapy option as the first line for weight loss and treatment of comorbid diseases. Further long-term studies are needed to confirm these results.

## Introduction

Obesity is a global health issue that contributes to a variety of disorders, including diabetes mellitus (DM), hypertension (HT), dyslipidemia, cardiovascular diseases, and respiratory diseases [1]. Bariatric surgery is the most effective treatment option for obesity compared to conventional medical treatment methods [2,3]. Bariatric surgical procedures are difficult to perform in patients with a BMI ≥ 50 kg/m^2^ due to massive hepatomegaly, limited intraabdominal working space, thick abdominal wall, and increased intraabdominal adipose tissue [4]. Surgical treatment is associated with high mortality, morbidity, and increased surgical risk in this patient group [5,6].

Sleeve gastrectomy (SG) is preferred to Roux-en-Y gastric bypass (RYGB) because it is clinically easier to perform, requires a shorter hospital stay, and has lower morbidity. In patients with a BMI ≥ 50 kg/m^2^ who underwent SG, lack of sufficient weight loss may require secondary bariatric surgical procedures [5-7]. On the other hand, recent studies have shown that SG provides effective weight loss in patients with a BMI ≥ 50 kg/m^2^ and there is no need for secondary malabsorptive procedures [8-10]. The appropriate bariatric surgery in patients with a BMI ≥ 50 kg/m^2^ is still controversial. In this study, we aimed to investigate the effect of SG on weight loss and associated comorbidities in patients with a BMI ≥ 50 kg/m^2^.

## Materials and methods

The study was approved by the Biruni University ethics committee. The prospectively maintained data obtained from patients with a BMI ≥ 50 kg/m2, who underwent SG between February 2016 - February 2020, were evaluated. All patients were operated on by the same bariatric surgeon. A multidisciplinary team comprising of a bariatric surgeon, dietician, endocrinologist, cardiologist, anesthesiologist, and psychiatrist assessed the patients preoperatively. Upper gastrointestinal endoscopy and abdominal ultrasonography were performed on all patients before surgery. All of the patients received low molecular weight heparin prophylaxis the night before surgery, which was also maintained in the postoperative period. The patients were assessed concerning demographic data (age, sex), anthropometric measurements (weight, height, BMI), comorbid diseases, biochemical parameters (lipid profile, glycated hemoglobin (HbA1c), fasting blood sugar), the weight loss in the 3^rd^, 6^th^, 12^th^, 18^th^, and 24^th^ months as well as the percentages of excess weight loss (EWL) and complications.

BMI equivalent to 25 kg/m^2^ according to BMI calculated by weight (kg)/height (m^2^) formula was defined as ideal body weight, while the difference between initial weight and ideal weight was defined as excess weight.

The percentage of EWL ( EWL%) = ((initial weight - current weight) / (initial weight - ideal weight)) × 100. The percentage of total weight loss (TWL%) = ((starting weight - current weight / starting weight)) × 100 were the formulas used [11].

The success of bariatric surgery was assessed according to the modified Reinhold criteria (as seen in Table 1) and Brion criteria [12-14]. According to the Brion criteria, surgical success in patients with BMI ≥ 50 kg/m² was defined as having a postoperative BMI ˂40.

**Table 1:** Modified Reinhold classification

Result	BMI (kg/m²)	Excessive Weight Loss %
Excellent	˂30	˃75
Good	30-35	50-75
Failure	˃35	˂50

The American Diabetes Association guidelines were used to make the diagnosis of DM [15]. DM was defined as HbA1c ≥ 6,5% or fasting blood glucose ≥126 mg/dl. Patients without medication with HbA1c levels of ˂ 6% were defined as resolved from diabetes and patients with HbA1c levels lower than preoperative HbA1c levels were defined as improved. Hypertension was defined as blood pressure (BP) >140/90 mm Hg. Remission was defined if the patient had normal BP (≤120/80) without any antihypertensive medications, and improvement was considered if the number of antihypertensive medications or dose of the antihypertensive medications was lowered. Total cholesterol levels were considered normal if <200, borderline high if between 200-239, and high if ≥240. Low density lipoprotein (LDL) levels were considered normal if ˂100, borderline high if between 130-159, and high if ≥160 high. Gastroesophageal reflux disease (GERD) severity symptom questionnaire was applied to patients with symptoms or complaints of GERD [16]. Patients with a severity symptom score above 4 or regular proton pump inhibitor (PPI) use, who showed hiatal hernia in their endoscopy, also underwent posterior hiatus repair. Remission was defined as the patient's absence of symptoms without the use of a PPI. An improvement was considered if the patient required a decrease in the dose of PPI or decrease in symptoms [16,17].

Surgical procedure

All surgeries were performed laparoscopically. The first trocar was introduced using visiport (between umbilicus and xiphoid with 1/3 proximity to the umbilicus); five trocars were used in total. The greater curvature of the stomach was de-vascularized. The short gastric vessels and gastrosplenic ligaments were divided using a LigaSure® device (Medtronic Parkway, Minneapolis, MN, USA). The stomach was transected by starting 2-4 cm proximally from the pylorus, until the gastroesophageal junction was reached. A 38 fr bougie were used. The first stapler used was an endo GIA™ 60 mm black tri-stapler (Medtronic Parkway, Minneapolis, MN, USA). The consequent staplers used were endo GIA™ 60 mm purple tri-staplers. Suture reinforcement was not performed on the stapler line. An intraoperative methylene blue stress leak test was routinely performed. A 10-mm Jackson-Pratt drain was routinely inserted along the suture line in all patients. All of the patients were started on liquid nutrition at the post-operative 24^th^ hour.

Statistical analysis

The current study was planned from the outset to increase validity and reliability. For descriptive statistics, the mean and SD values were used if the variables were continuous, while the median and percentage values were used for discrete variables. The normality of the variables was analyzed using the Kolmogorov-Smirnov test. Statistical comparisons between groups were performed using the repeated ANOVA test followed by the post hoc Bonferroni test. Two-sided p values were considered statistically significant at p≤0.05. All statistical analyses were carried out by using the R software/programming (version 3.6.2 (2019-12-12) - CRAN).

## Results

A total of 138 patients were operated on; 104 (75.36%) patients were female. All operations were performed laparoscopically. The mean age was 37.41 ± 12.33, the mean BMI was 56.36 ± 7.661, and the mean excess weight was 82.95 ± 21.15. There were 44 (31.88%) patients with a BMI ≥ 60. Ninety (65.21%) patients underwent SG, Twenty-three (16.66%) patients underwent SG + cholecystectomy, 21 (15.21%) patients underwent SG + hiatal hernia repair (HHR) and four (2.89%) patients underwent SG + HHR + cholecystectomy. The baseline characteristics, obesity-related comorbidities, surgery types, and laboratory parameters in this study are shown in Table 2.

**Table 2:** Patient characteristic BMI: body mass index; SG: sleeve gastrectomy; HHR: hiatal hernia repair; DM: diabetes mellitus; OSAS: obstructive sleep apnea syndrome; GERD: gastroesophageal reflux disease; HbA1c: glycated hemoglobin.

	n (%)	
Total Number of Patients	138 (100)	
Female	104 (75.36)	
Age	37.41±12.33	
Height	162.7± 8.783	
Weight	149.3±24.16	
BMI	56.36±7.661	
Excess Weight	82.95±21.15	
Surgery Type		
-Sleeve Gastrectomy	90 (65.21)	
-SG + Cholecystectomy	23 (16.66)	
-SG + HHR	21 (15.21)	
-SG + HHR +Cholecystectomy	4 (2.89)	
Hospital Stay (median day)	3 (3-6)	
Hypertension	44 (31.88)	
Type 2 DM	47 (34.05)	
Cardiac Disease	7 (5.07)	
OSAS	13 (9.42)	
Respiratory Disease	25 (18.11)	
GERD	25 (18.11)	
Psychological Disorders	8 (5.79)	
	Before Surgery	After Surgery
HbA1c	6.10±1.498	5.20±0.505
-HbA1c (Diabetic Patients)	7.41±1.885	5.482±0.687
Glucose	114.4±36.27	88.80±11.08
Total Cholesterol	190.9±41.68	191.2±41.98
LDL	123.2±30.66	122.5±36.22

Weight loss

The mean BMI, EWL%, and TWL% of the 0^th^, 3^rd^, 6^th^, 12^th^, 18^th^, and 24^th^ months, respectively, are provided in Table 3.

**Table 3:** Distribution of the mean BMI (kg/m2), EWL% and TWL% for patients in different months and the results of repeated ANOVA test followed by post hoc Bonferroni test The mean values of all months were found significantly different for EWL%, TWL% and BMI (p<0.001). According to the Bonferroni multiple comparison test results, all possible binary comparison results for the reference months (0^th^, 3^rd^, 6^th^, 12^th^, 18^th^, 24^th^) were significantly different for EWL%, TWL% and BMI variables (p<0.001), but EWL% (p = 0.527), TWL (p = 0.396) and BMI (p=0.657) were not found significantly different between the 18^th^ and 24^th^ months. BMI: body mass index; EWL: excess weight loss; TWL: total weight loss; ANOVA: analysis of variance.

Time (month)	n	BMI	±SD	P	EWL%	±SD	p	TWL%	±SD	p
0	138	56.36	7.660	<0.001*			<0.001^*^			<0.001^*^
3^rd^	125	45.10	7.128		36.70	8.294		20.17	4.049	
6^th^	116	39.76	6.165		54.34	10.62		29.59	5.706	
12^th^	93	35.48	6.285		67.58	13.37		36.93	6.657	
18^th^	79	33.96	5.305		72.45	13.42		39.62	7.130	
24^th^	50	33.41	6.198		74.14	10.03		40.65	8.696	

**Figure 1:**
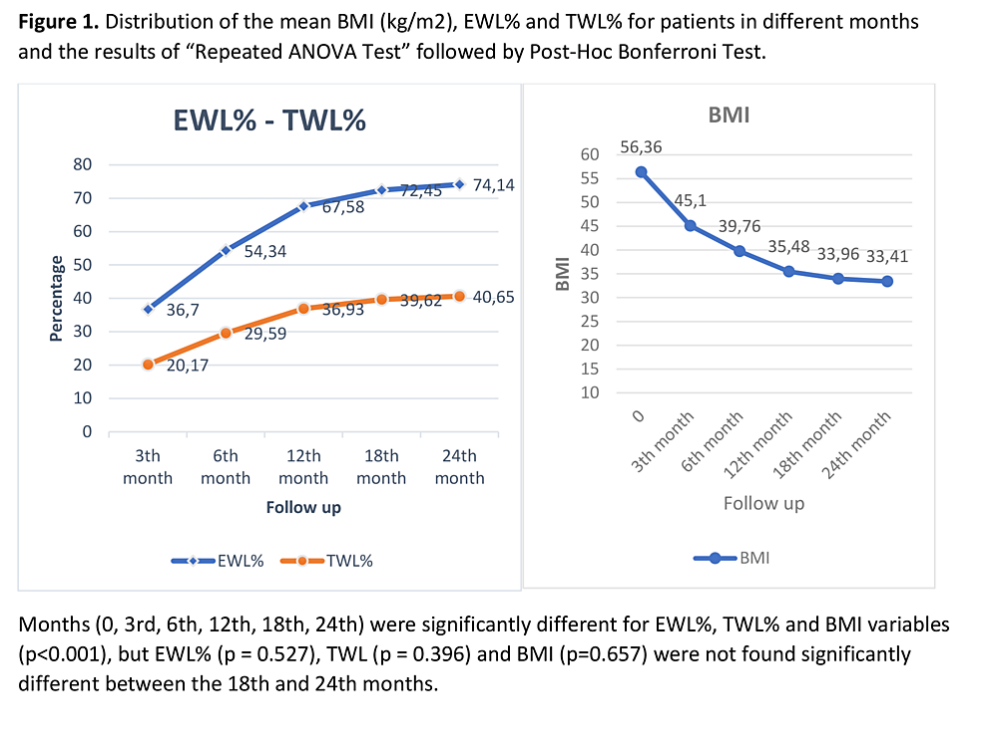
Distribution of the mean BMI (kg/m2), EWL% and TWL% for patients in different months and the results of repeated ANOVA test followed by post hoc Bonferroni test Months (0, 3^rd^, 6^th^, 12^th^, 18^th^, 24^th^) were significantly different for EWL%, TWL% and BMI variables (p<0.001), but EWL% (p = 0.527), TWL (p = 0.396) and BMI (p=0.657) were not found significantly different between the 18^th^ and 24^th^ months. BMI: body mass index; EWL: excess weight loss; TWL: total weight loss; ANOVA: analysis of variance.

In months 12^th^, 18^th^, and 24^th^, the mean EWL% was found to be 67.58%, 72.45%, and 74.14%, the mean BMI was 35.48, 33.96, and 33.41, and the mean TWL% was found to be 36.93%, 39.62% and 40.65% ( Figure 1).

**Figure 2:**
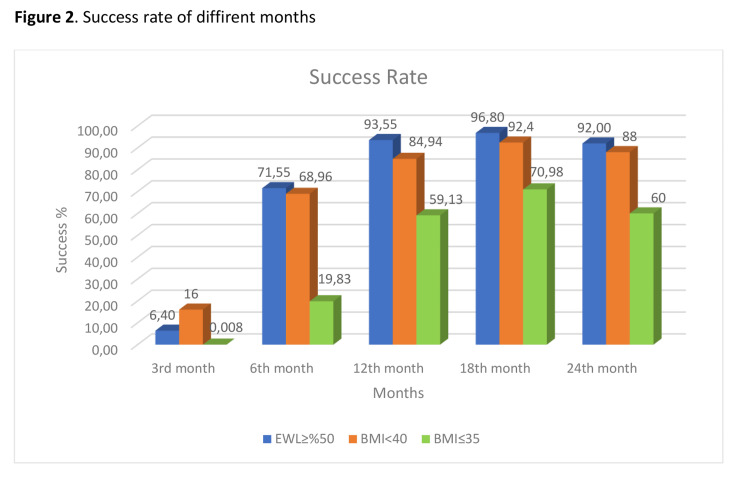
Success rate of different months BMI: body mass index; EWL: excess weight loss.

The mean values of all months were found significantly different for EWL%, TWL%, and BMI (p<0.001). According to the Bonferroni multiple comparison test results, all possible binary comparison results for the reference months (0^th^, 3^rd^, 6^th^, 12^th^, 18^th^, 24^th^) were significantly different for EWL%, TWL%, and BMI variables (p<0.001), but EWL% (p = 0.527), TWL (p = 0.396) and BMI (p=0.657) were not found significantly different between the 18^th^ and 24^th ^months. The success rate was found to be 93.55% in the first year and 92% in the second year when EWL being considered as ≥50%. With the success rate for BMI being considered as ≤35, it was found to be 59.13% in the first year and 60% in the second year. When the BMI being considered was <40, it was found to be 84.94% in the first year and 88% in the second year (Table 4, Figure 2).

**Table 4:** Postoperative success rate according to follow-up percent excess weight loss (EWL%), body mass index (BMI), Reinhold criteria

	%EWL				BMI				
Follow-up	EWL< 50^a^ n (%)	EWL50–74^b^ n (%)	EWL≥75^c^ n (%)	Good and Excelent^b,c ^ %	<30^a^ n (%)	30-35^b^ n (%)	>35^c^ n (%)	Good and Excelent^a,b ^ %	BMI˂40 n(%)
3^rd^ Month	117/125 (93.60)	8/125 (6.4)	-	6.4	-	1/125 (0.008)	124/125 (0.992)	0.008	20/125 (16)
6^th^ Month	33/116 (28.45)	78/116 (67.24)	5/116 (4.310)	71.55	3/116 (2.586)	20/116 (17.24)	93/116 (80.17)	19.83	80/116 (68.96)
12^th^ Month	6/93 (6.452)	51/93 (54.84)	36/93 (38.71)	93.55	17/93 (18.27)	38/93 (40.86)	38/93 (40.86)	59.13	79/93 (84.94)
18^th^ Month	3/79 (3.797)	38/79 (48.10)	38/79 (48.10)	96.8	25/79 (31.65)	31/79 (39.24)	23/79 (29.11)	70.89	73/79 (92.4)
24^th^ Month	4/50 (8.0)	20/50 (40.0)	26/50 (52.0)	92.0	16/50 (32.00)	14/50 (28.00)	20/50 (40.00)	60.00	44/50 (88)

No mortality or major morbidity such as stapler line leaks, gastrointestinal bleeding, or intraabdominal bleeding was observed in the first 30 days. One patient developed tetany due to hypocalcemia, which improved with intravenous calcium and vitamin D treatment. Fever and gastroenteritis developed in one patient and were resolved with treatment. Deep vein thrombosis or pulmonary embolism was not observed.

Resolution of comorbidities

While complete remission of type 2 diabetes was observed in 42 (89.36%) patients, it was observed that five patients had shown improvement. In diabetic patients, the mean pre-operative HbA1c was 7.41±1.885 while the mean post-operative HbA1c was 5.482±0.687 (p=0,00). Complete remission was observed in 13 patients with obstructive sleep apnea syndrome (OSAS); they stopped using continuous positive airway pressure equipment. Improvement was observed in only five out of eleven patients with hyperlipidemia; no remissions were observed. Complete remission of HT was observed in 41 patients and three patients showed improvement. The reflux symptoms of 25 patients, who had reflux complaints and underwent hiatal hernia repair, completely disappeared.

## Discussion

Nowadays, SG is the most common bariatric surgical procedure in the world due to its technical ease compared to other bariatric procedures [18,19]. SG was developed as the first stage of a two-stage bariatric surgical procedure, especially in patients with a BMI ≥ 50 kg/m^2^ [6,20]. Subsequent studies have proven that SG alone is also an effective bariatric surgical procedure [8,21,22]. In some studies, it has been stated that SG provides less weight loss and improves less insulin sensitivity compared to RYGB in the long term [23,24]. On the other hand, recent randomized clinical studies showed that SG and RYGB were equally effective in weight loss and the treatment of comorbidities [25,26].

One of the most important methods used to assess the success of surgery is EWL%. In our study, we found that the mean EWL% was 67.58±13.37 in the 12^th ^month and 74.14± 10.03 in the 24^th^ month. The mean values of all months were found significantly different (p<0.001). According to the Bonferroni multiple comparison tests, in all possible binary comparisons (3^rd^, 6^th^, 12^th^, 18^th^, 24^th^), EWL% were significantly different (p<0.001), except for the 18^th^ and 24^th^ months. It was observed that the process of weight loss continued until the 24^th^ month, but there was no statistically significant difference between months 18 and 24. The success rate was 93.55%, 96.8%, and 92% at the 12^th^, 18^th,^ and 24^th^ months, respectively, when EWL% being considered was ≥50, according to the Modified Reinhold criteria. When assessed according to the Biron criteria (BMI˂40), it was found to be 84.92%, 92.4%, and 88% in the 12^th^, 18^th, ^and 24^th ^months.

In the study by Bhandari et al., which included 514 in-patients with a BMI ≥ 50 kg/m^2^, the EWL% of the 2nd and 3^rd^ year was found as 74.24% and 62.38% in the SG group, respectively, 71.4% and 69.55% in the RYGB group respectively. In the same study, this rate was given as 87.88% and 85.11% in the banded SG group [26]. Gil-Rendo A et al. performed a study on 134 patients who underwent SG and their EWL% in years 1 and 2 were 61.3%, and 62.6%, respectively [27]. In a study conducted by Bettencourt-Silva R et al. with 213 in-patients with a BMI ≥ 50 kg/m^2^, the group that underwent SG had EWL% of 58.74 and 59.90 in years 1 and 2, respectively. In the RYGB group, these were 67.58 and 72.19, respectively [28]. In a study performed by Arapis K et al. which included 210 in-patients with a BMI ≥ 60 kg/m^2^, the EWL% was 48.81 and 54.17 in the SG group in years 1 and 2, respectively. In the RYGB group, these were 53.96 and 60.64, respectively [10]. Celio AC et al. conducted a study with 50,987 in-patients with a BMI ≥ 50 kg/m2; the EWL% was 49% and %58 in the SG and RYBG group in the first year, respectively [29]. In a study conducted by Uno K et al. consisting of 48 super obese patients, the EWL% was reported as 57.7% and 65.1% in the SG group in years 1 and 2, respectively; in the RYGB group, it was reported as 73.4% and 73.7%, respectively [30].

In the meta-analysis study of Wang Y et al. including 12 studies, RYGB was found to be superior in terms of EWL% in the first 12 months, while this situation was found to be equal for SG and RYGB in the 24^th^ month [31]. Similarly, in the study of Bhandari et al., it was stated that RYGB and SG had similar averages in terms of EWL% in the 3^rd^ year [26]. SG was recommended as the primary surgical procedure in the study by Arapis K et al., involving the in-patients with a BMI ≥ 60 kg/m^2^. The same study stated that SG and RYGB gave similar results in the 4^th^ year in terms of EWL% and BMI changes [10].

In the randomized Swiss Multicenter Bypass or Sleeve Study (SM-BOSS) study comparing bariatric surgery patients who underwent SG and RYGB recently, it was observed that there was no significant difference between SG and RYGB groups in terms of weight loss. Excessive BMI loss was similar between LSG and LRYGB at each time point (1 year: 72.3±21.9% vs. 76.6±20.9%, P =0.139; 2 years: 74.7±29.8% vs. 77.7±30%, P = 0.513; 3 years:70.9±23.8% vs. 73.8±23.3%, P =0.316) [25]. In a study conducted by Mulita et al. which included 209 patients who underwent SG and six years follow-ups, the median % EWL at 1, 2, 3, 4, 5, and 6 years postoperatively was 80.9%, 79.1%, 73.8%, 71.8%, 71.5%, and 64.9%, respectively. In the same study six years post-surgery, deficiencies of hemoglobin, ferritin, and B12 worsened; whereas, there was no significant difference in deficiencies of iron, folic acid, magnesium, and phosphorus [32].

Significant improvements were observed in comorbid diseases in the operated patients. While complete remission and recovery were observed in comorbidities such as DM, HT, OSAS, and GERD, complete remission was not seen in patients with hyperlipidemia. It was found that only 45.45% of patients with hyperlipidemia improved. There are studies which state that better results are obtained with RYGB in the improvement of comorbid diseases, especially type 2 DM [31]. A recent meta-analysis comparing SG and RYGB, which included 18,455 patients and 62 studies to assess obesity-related comorbidities, found that RYGB had a statistically significant superiority in the remission of hyperlipidemia and GERD. However, no statistically significant differences were seen in the DM and OSAS remission [33]. In the study conducted by Singla V et al. which included 75 super obese patients, the remission rate in Type 2 DM was found to be 85.7% in the SG group and 77.77% in the OAGB group (p = 0.59) [34] (Table 5).

**Table 5:** Studies of patients with a BMI ≥ 50 kg/m² BMI: body mass index; EWL: excess weight loss; SG: sleeve gastrectomy; RYGB: Roux-en-Y gastric bypass; OAGB: one-anastomosis gastric bypass.

Study Name and Year	Number of Patients	BMI	EWL% YEAR 1	EWL% YEAR 2	Mortality - Major Complications
Arapis K et al. (2018) [10]	SG (91)	68.2±7.1	48.81±5	54.17±5	1.09% - 16.1%
	RYGB (119)	65.1±4.3	53.96±5	60.64±5	0.84% - 26%
Gil-Rendo A et al. (2019) [27]	SG (134)	55.9±6.7	61.3±18	62.6±22.7	3.7% - 15.7%
Bettencourt-Silva R et al. (2018) [28]	SG (67)	54.73±4.89	58.74±17.7	59.90±18.15	0% -6.6%
	RYGB (127)	54.32±4.28	67.58±14.26	72.19±14.62	
Celio AC et al. (2016) [29]	SG (8868)	57.8	49±15.7		0.2% - 11.1%
	RYGB (42119)	57.3	58±14.5		0.3% - 11.5%
Uno K et al. (2017) [30]	SG (28)	57.1±5.1	57.7±21.4	65.1±23.4	0 - 10.7%
	RYGB (20)	55.7±4.2	73.4±16.1	73.7±22	0 - 20%
Singla V et al. (2019) [34]	SG (50)	54.18±4.06	56.2±18.92		0 - %4
	OAGB (25)	53.76±3.28	74.57±13.24		0 - 0
Gonzalez-Heredia R et al. (2016) [35]	SG (77)	64.9±4.2	43.6±13.8	45.8±19.2	0 - 2.2%
	RYGB (12)	64.2±2.5	61.4±18.4	68.5±16.8	0 - 0

In the study by Bettencourt-Silva et al., it was observed that there was no difference in diabetes remission in the 1st and 2nd year between RYGB, SG, and AGB. (P = 0.91-p = 0.13) [28].

Apart from weight loss, different pathophysiological mechanisms play a role in the recovery of comorbidities after SG. These include mechanisms such as increased gastric emptying and intestinal transit, increased glucagon-like peptide-1 ( GLP-1) hormone level, and decreased ghrelin levels [36,37]. In our study, remission was observed at a rate of 89.36% (n = 42) in type 2 DM patients. While the mean HbA1c of diabetic patients in the preoperative period was 7.00 (5.4-14.7), it was found to be 5.4 (4.2-8.1) postoperatively. Remission was observed in all OSAS patients. GERD after SG is an important problem. The patients that had reflux symptoms and hiatal hernia before surgery also underwent concomitant hiatal hernia repair. In all of these patients, the reflux symptoms disappeared in the post-operative period. SG can be performed more easily and safely than other surgical procedures given the large liver volume, limited intraabdominal operating space, increased abdominal wall thickness, and increased abdominal fat tissue [38].

Since comorbid diseases are more common in patients with a BMI ≥ 50 kg/m^2^, complication and mortality rates are also high [27,29,39,40]. As the risk of mortality is high when in-patients with a BMI ≥ 50 kg/m^2 ^develop complications, surgeries need to be performed with minimal complications. When complications develop in such patients, it is more difficult to intervene as compared to other patients. This is another factor affecting mortality. As per some studies performed, the rate of complications such as stapler line leaks, stricture, intraabdominal hemorrhage, abscess, pulmonary embolism, deep vein thrombosis, pneumonia, myocardial infarction, and wound infection is in the range of 3.8-15.7%. The duration of surgery and hospital stay are also relatively long [5,27,29,41,42]. According to the studies conducted, the mortality rate was in the range of 0.008%-0.18% in non in-patients with a BMI ≥ 50 kg/m^2 ^while the in-patients with a BMI ≥ 50 kg/m^2^ had mortality rates ranging up to 3.7% [27,29,40,41,43-45].

No mortality and major complications were observed in our study. Concomitant surgeries such as cholecystectomy and HHR had no effect on mortality and morbidity. One of the major complications that may be observed during SG surgery is stapler line leaks. The possibility of a leak, as a result of a technical error, was checked via methylene blue leak test performed during surgeries. It was ensured during surgery that the stapler line was straight and there were no twists in the stomach. To prevent leakage, due to increased intraluminal pressure in the postoperative period, the patients were advised not to drink suddenly and quickly while consuming fluids, but to rather take smaller sips slowly [46]. 

## Conclusions

Our study has shown that SG is a rather effective method on its own for weight loss and resolution of comorbidities in patients with a BMI ≥ 50 kg/m². It can be performed in experienced centers with minimal morbidity and no mortality. Considering the large liver volume, limited intraabdominal working space, and increased abdominal wall thickness in patients with a BMI ≥ 50 kg/m^2^, SG should be considered as the first option. Long-term prospective randomized studies are needed to confirm these findings.
